# Oral health in a group of patients with Rett syndrome in the regions of Valencia and Murcia (Spain): A case-control study

**DOI:** 10.4317/medoral.19743

**Published:** 2014-09-30

**Authors:** María C. Fuertes-González, Francisco J. Silvestre

**Affiliations:** 1 Dental surgeon of the Red Cross Special Patients Dental Clinic, Valencia. Professor of the Master of Hospital Odontology and Special Patients. Department of Stomatology, University of Valencia; 2 Assistant Professor of the Department of Stomatology (University of Valencia). Stomatology Unit, Dr. Peset University Hospital Valencia, Spain

## Abstract

Objectives: Rett syndrome (RS) is a rare disease with oral manifestations that have not been described in detail or in a standardized manner in the literature. The present study describes the oral health of the population with RS in two Spanish regions, following the protocol of the World Health Organization for conducting common oral health surveys. Study Design: A prospective, observational case-control study was carried out, involving a group of patients with RS (n1=41) and a mean age of 13.37±3.19 years, and an age- and gender-matched control group without RS (n0=82). The data referred to oral health and habits were recorded by means of a questionnaire and oral examination was used to document caries indicators (prevalence of caries, df(t), df(s), DMF(T), DMF(S) and indices referred to dental loss, morbidity, restoration), the Community Periodontal Index (CPI), and the most characteristic oral manifestations. Results: The most frequent oral habit in the patients with RS was diurnal bruxism, followed by stereotyped tongue movements and oral breathing. The caries scores were lower in the RS population than in the control group, but patients with RS showed greater periodontal alterations and a greater prevalence of drooling, dental wear, high-arched palate and anterior open bite. Conclusions: The population with RS exhibits characteristic and early oral habits and alterations, and periodontal problems that are more notorious than caries disease, so that our efforts should focus on the diagnosis and early correction of the parafunctional habits, promoting restorative treatment, and providing instructions on correct oral hygiene.

** Key words:**Rett syndrome, oral habits, bruxism, caries.

## Introduction

Rett syndrome (RS) is a chromosome X-linked (mutation of the MECP2 gene located in Xq28) neurological development disorder almost exclusively found in females. It is characterized by psychomotor development regression with autistic manifestations, deceleration of cephalic growth, seizures, and stereotyped repetitive movements of the hands (1,2).

Rett syndrome is one of the so-called rare diseases, whose estimated prevalence is four cases per 100,000 inhabitants. Application of this rate to the Spanish population indicates that not all cases of RS are adequately recognized and registered. In effect, at a time (in the year 1999) in which the official Spanish population was 39,580,600 inhabitants, a total of 207 cases of RS were documented (3) – this being a very low number of cases.

Identification of the MECP2 mutation is not strictly necessary for establishing a diagnosis of RS (4), which continues to be largely based on clinical criteria (1). This has given rise to difficulties in establishing a differential diagnosis with other autistic spectrum disorders (ASDs) (5).

Regarding the dental literature, few articles on RS can be found, and data collection moreover has not been standardized (6-12). The existing descriptions refer to oral manifestations and habits that are common to other clinical conditions characterized by seizures, difficulties in performing correct oral hygiene, walking problems and/or excessive oral/digital-manual habits. Nevertheless, it can be suggested that bruxism is the oral habit most commonly associated to RS (13).

The only information available on the oral health of patients with RS comes from articles with a very low level of scientific evidence, and there are no case-control studies comparing patients with and without RS. The present study was therefore designed to characterize this rare disease, based on all the documented patients in two Spanish regions, and establishing a standardized registry of their oral manifestations and habits, following the recommendations of the World Health Organization (WHO) for the conduction of oral health surveys.

## Material and Methods

-Study design

A prospective, observational case-control study was carried out, using a questionnaire and direct oral examination to analyze the main variables and features defining oral health, followed by a comparative study between patients with RS and a control group without RS.

-Study sample

The study population comprised the group of females with RS ascribed to the Spanish Rett Syndrome Association (*Asociación Española de Síndrome de Rett*, AESR) and residing in the region of Valencia or Murcia. The inclusion criteria were: an established clinical diagnosis of RS, ascription to the AESR, and residency in the region of Valencia or Murcia. The AESR currently has a total of 159 members in Spain, including 54 patients from the two aforementioned regions. Of these subjects, 5 did not present a clinical diagnosis of RS, while in 8 cases the parents and/or tutors did not give consent to participation in the study. Consequently, the study sample (n1) consisted of 41 patients with RS ascribed to the AESR and corresponding to the region of Valencia or Murcia (34%, 34%, 17% and 15% from the provinces of Valencia, Alicante, Castellón and Murcia, respectively). The patient age ranged from 2-37 years (mean age 13.37±3.19), and all were females. The oral examinations were carried out in the Red Cross Special Patients Dental Clinic (Valencia).

The control group in turn consisted of twice as many subjects as the group of patients with RS (n0=82 subjects without RS) randomly chosen. The inclusion criteria were: female gender, accompanying person of the patients with residency or census inscription in the region of Valencia or Murcia, age matched to the study group, and the absence of systemic diseases and/or chronic drug treatment (anamnesis). The examinations in the control group were centralized and were carried out in a dental clinic in Valencia and another in Murcia.

-Methodology

The examiner first administered a questionnaire based on a direct interview of the parents and/or tutors, with systematic documentation of the case history, oral habits and oral hygiene, and previous dental treatments. An informative sheet was also provided along with the informed consent form (photographs included). A single examiner (CFG) previously calibrated for the study (Kappa index 0.961, *p*<0.001) then carried out the oral examination of the patient.

**Figure 1:**
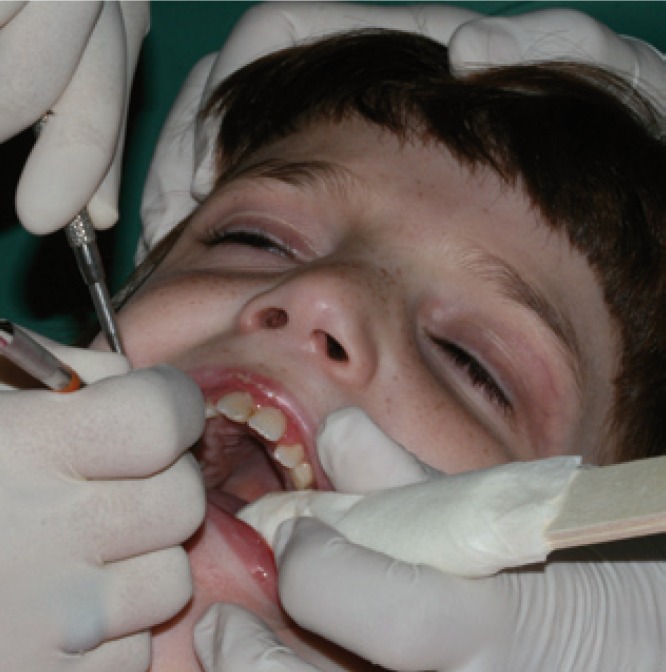
Oral aperture and positioning for the examination of a patient with Rett syndrome.

The material used for the examination consisted of a standard periodontal probe and a flat intraoral mirror (no. 5). Examination in uncooperative RS patients was carried out with control of the head and hands by the auxiliary personnel and parents and/or relatives of the patient. Use was made of oral retractors produced in the clinic and consisting of the handle of an alginate or plaster mixing spatula wrapped in gauze and covered with tape to make it more impermeable. The device was placed between the arches on one side of the oral cavity while the other side was explored (Fig. 1).

In relation to the study variables, we recorded oral habits such as bruxism (diurnal/nocturnal), stereotyped tongue movements, oral breathing, and tongue and/or lip interpositioning. All dental surfaces were examined and the WHO criteria were employed for diagnosis and coding (14). As caries indicators we recorded the prevalence of caries in the temporal dentition (TD) and permanent dentition (PD), as well as total caries, and the DMF(T) index, DMF(S) index, df(t) index, df(s) index, dental morbidity index (“d” component of df(t)/df(t) and “D” component of DMF(T)/DMF(T), expressed as a percentage), restoration index (“f” component of df(t)/df(t) and “F” component of DMF(T)/DMF(T), expressed as a percentage) and dental loss index (“M” component of DMF(T)/DMF(T), expressed as a percentage). Periodontal health in turn was evaluated using the Community Periodontal Index (CPI), scored as follows: 0 = healthy, 1 = bleeding observed directly or using the intraoral mirror, after the examination, 2 = tartar observed during the examination, 3 = pocket depth 4-5 mm, and 4 = pocket depth ≥ 6 mm. Lastly, the examination was completed by recording the presence or absence of drooling, posterior crossbite, anterior open bite, high-arched palate and the degree of dental wear assessed by the modified O’Brien dental erosion index (15): 0 = no wear facets, 1 = enamel wear facets, 2 = enamel and dentin wear facets, and 3 = dentin wear facets close to the pulp.

-Data analysis

The data were analyzed using the SPSS version 18.0 statistical package. A descriptive study was made, reporting the means and percentages, and corresponding 95% confidence intervals (95%CI). The bivariate comparative study of means was carried out using the Student t-test and analysis of variance (ANOVA), while the comparison of proportions was based on the chi-squared test (χ²) or Fisher exact test when the sample size was too small and the criteria for applying the chi-squared test were not met. Statistical significance was considered for *p*<0.05.

## Results

In the RS group (n1=41), 14 patients (34.14%) had no documented genetic alteration, while in 27 cases (65.85%) a molecular diagnosis confirming the clinical diagnosis was available. Most of the patients with RS (75.6%) received some kind of medication for control of the syndrome, such as anxiolytic agents and/or antipsychotics. The most frequently prescribed medication was an antiepileptic (valproic acid, administered to 36.58% of the patients). As regards the clinical manifestations associated to the syndrome, the most common (95.12%) were manual stereotypias or repetitive movements of the hands – generally a rubbing movement on the body midline. Although only 22% of the patients were able to use a few words, 87.8% showed signs of being able to recognize relatives and people close to them. On the other hand, 65.85% of the patients with RS suffered epilepsy, were able to walk with assistance, and experienced laughing crises for no apparent reason. Forty-three percent had hyperventilation episodes, and 39.02% suffered apnea. Both of these manifestations could be found in one same patient. As regards the orthopedic problems most commonly associated with the disease, 12.19% of the patients with RS had undergone surgery for the correction of scoliosis, and 2.43% for the correction of tiptoe walking.

Comparison of the oral hygiene habits in patients with RS versus the controls is shown in  Table 1. All of the patients with RS that brushed their teeth did so with help (95.1%). Brushing was manual in 73.2% of the cases, 36.6% brushed their teeth once a day and 41.5% wetted the brush in an oral rinse. In the control group, the percentage of patients that brushed their teeth was 97.5%, and in most cases (89%) brushing required no help, and involved the use of toothpaste (70.7%).

**Table 1:**
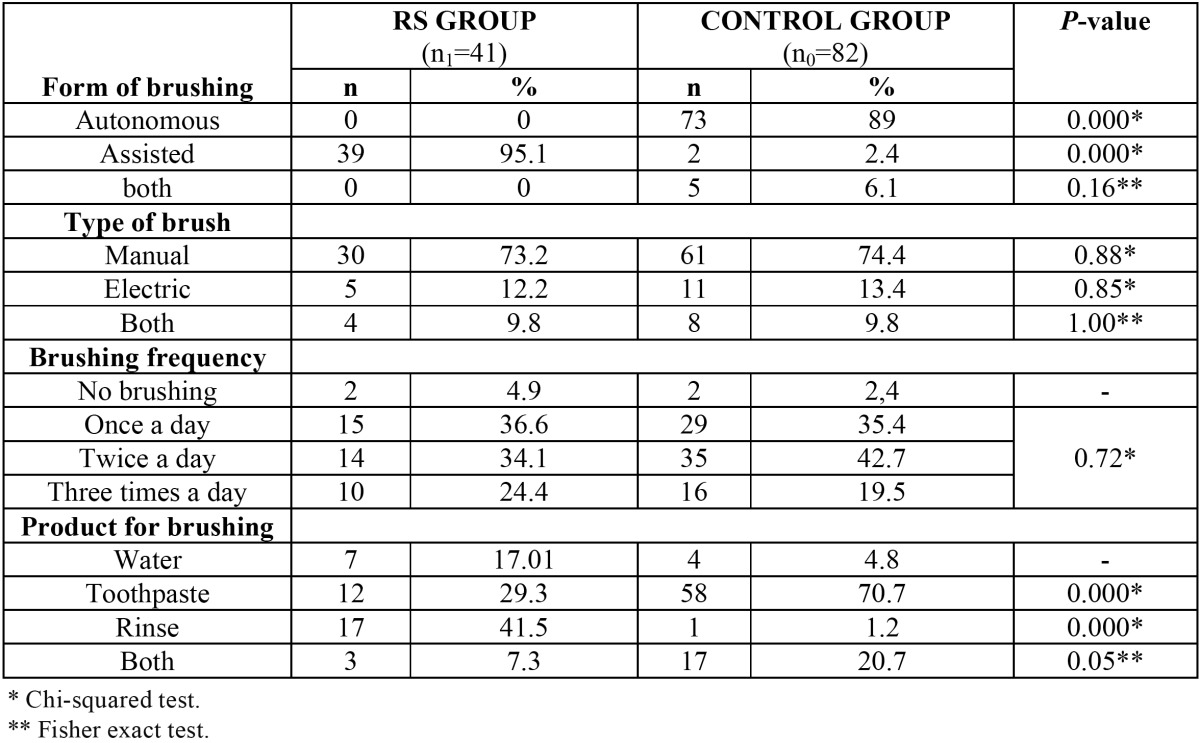
Comparison of oral hygiene habits between patients with Rett syndrome and the control group.

A total of 63.4% of the patients with RS had undergone dental checks. Tartrectomy had been performed in 24.4%, fillings in 9.8%, and extractions in 19.5%. General anesthesia was used for dental treatment in 14.6% of the cases. On contrasting dental care between the two groups, 75.6% of the controls were seen to have undergone dental checks, with tartrectomy in 20.7%, fillings in 53.7%, and extractions in 31.7%. Dental treatment was carried out under general anesthesia in 6.1% of the controls. The only significant difference between the two groups corresponded to the number of fillings (*p*<0.001).

 Table 2 shows the existence of statistically significant differences in the prevalence of all oral habits between the two groups of the study. The most frequent oral habit in the patients with RS was bruxism (73.2%), with the distinction of two groups: diurnal (68.3%) and nocturnal (4.9%). In turn, 56.1% showed stereotyped tongue movements, i.e., repetitive tongue thrusting and/or twisting with no functional purpose. Oral breathing was observed in 34.1% of the sample, lip interpositioning in 22%, and tongue interpositioning in 9.8%. Bruxism was present in only 32.9% of the controls (diurnal 9.7% and nocturnal 23.2%), with no stereotyped tongue movements, and the prevalence of oral breathing and lip and/or tongue interpositioning was significantly lower than among the patients with RS (7.8%, 2.4% and 1.2% respectively).

**Table 2:**
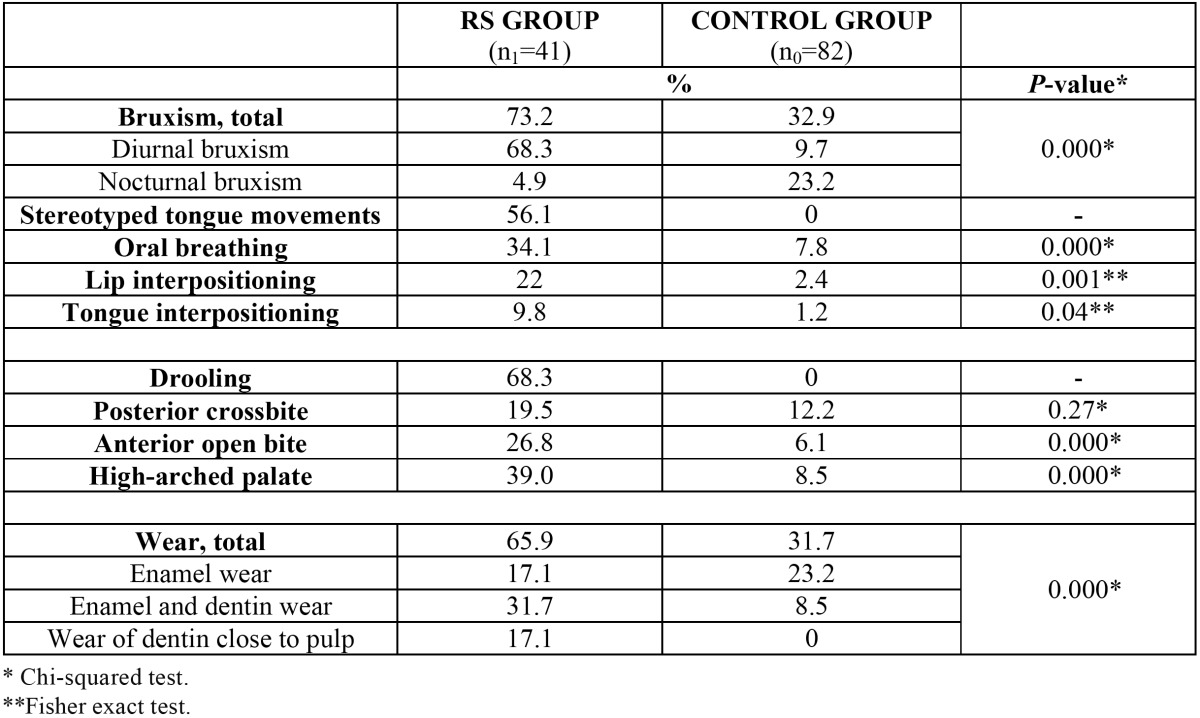
Comparison of oral habits and manifestations between patients with Rett syndrome and the control group.

The results referred to the caries indicators and periodontal health are shown in  Table 3. Statistically significant differences were observed in the prevalence of total caries and caries in PD (*p*=0.005 and *p*=0.003 respectively), in the “f” (filling) component of the df(t) index and df(s) score (*p*<0.001), and in the DMF(T) and DMF(S) scores (*p*=0.007 and *p*=0.009, respectively) – again at the expense of the “F” component (*p*=0.002). In all these cases the scores were higher in the control group than in the group with RS. In contrast, the morbidity index was significantly higher in the group with RS than in the control group, referred to both TD (*p*<0.001) and PD (*p*=0.03). The opposite applied in the case of the restoration index, with higher scores in the control group – statistical significance being reached in the case of TD (*p*<0.001). Lastly, the CPI score was significantly higher in the group with RS (1.65) than in the controls (0.74) (*p*<0.001).

**Table 3:**
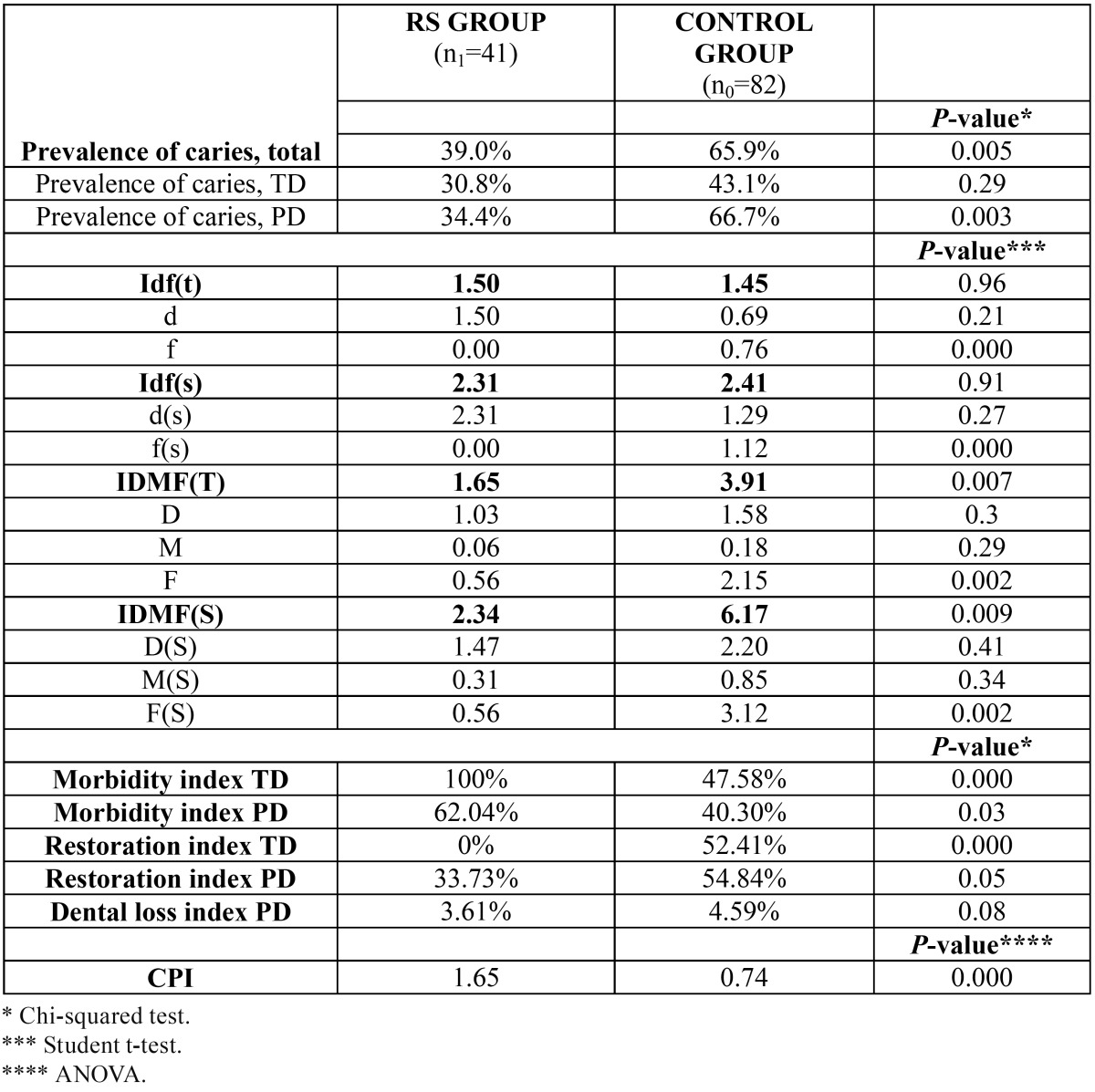
Comparison of caries indicators and periodontal health between patients with Rett syndrome and the control group.

Regarding the oral examination, drooling, anterior open bite and a high-arched palate were significantly more prevalent in the group of patients with RS, and the severity of dental wear was also greater: in the control group only 31.7% of the patients presented dental wear, versus 65.9% of the patients with RS. The most common finding in the control group was wear limited to the dental enamel (23.2%), while the most frequent presentation among the patients with RS was involvement of both the enamel and dentin (31.7%) ( Table 2).

## Discussion

The dental literature has offered no specific recommendations on behavior guidance for the dental examination of patients with RS. The poor communication and important mental retardation of these subjects limits the psychological strategies, usually employed in patients with autistic spectrum disorders (ASDs) (16-19), to controlled physical measures applied in a relaxed and family environment, that was precisely the pattern that we follow to carry out the oral examination in this study.

Great similarities in oral hygiene habits are observed with autistic spectrum patients, where assisted manual tooth brushing is also the most common practice (20,21) - possibly due to the limit tolerance of the sound and/or vibration of electrical brushes in both types of patients.

Bruxism has been described in most dental publications on RS (8-12), and represents the most characteristic oral habit of the syndrome. However, we must look to the medical literature to find studies involving larger sample sizes (3,22-24), where a broad range in the prevalence of diurnal bruxism has been reported (53-95%). In the present study, diurnal bruxism was observed in 68.3% of the sample, which is consistent with the aforementioned range. In contrast, the percentage of nocturnal (or sleep) bruxism was substantially lower than reported by other authors (4.9% versus 27% and 55%) (25,26), and was more in line with those studies that have reported no patients with nocturnal bruxism (12). Other important habits in these patients were stereotyped tongue movements (56.1%) and oral breathing (34.1%) – all with prevalences similar to or even higher in the figures published in the literature (56.6% and 57.57%, respectively) (24,27).

To date, no studies have collected data in an objective and standardized manner to establish the levels of dental caries in RS. Of note is the observation that total caries were significantly more prevalent in the controls than in the patients with RS. No differences were found on comparing the caries indices in temporal dentition (TD), i.e., df(t) and df(s), between the two groups, with the exception of the “o” component, referred to restored teeth. It is therefore deduced that the controls receive more conservative treatments in TD than the patients with RS. In permanent dentition (PD), both the DMF(T) and the DMF(S) scores were far higher in the control group than in patients with RS – particularly at the expense of the “o” component, in the same way as in TD. Therefore, the index that possibly offers most information on the oral health of patients with RS compared with the general population is the restoration index. The data suggest that the levels of conservative dental treatments (fillings) are significantly lower among the patients with RS. This coincides with the results of Anders *et al.* (28), who found that while the prevalence of caries in mentally disabled patients was no greater than in the general population, the levels of untreated caries were considerably higher among the patients – probably because of the physical and behavioral management problems, lack of experience on the part of the dental professionals, and resource limitations.

As regards periodontal health, the mean CPI score in the group of patients with RS was 1.65, versus 0.74 in the control group. Such observations are consistent with the data found in the literature. In effect, studies of periodontal disease in mentally disabled patients (mental retardation not associated to Down syndrome and autism) uniformly report increased prevalence and severity of gingivitis and periodontitis secondary to the accumulation of dental plaque, compared with the general population (28). This may be explained by the difficulties parents and/or caregivers experience in providing adequate daily assisted tooth brushing.

Patients with RS do not present very specific oral manifestations, though they do tend to have a higher prevalence of drooling, high-arched palate and anterior open bite (8,10,12,23). Anterior open bite is a multifactorial phenomenon resulting from unfavorable growth patterns and incorrect mandibular positioning such as that derived from exacerbated oral breathing and tongue thrusting habits (29). Another characteristic of RS is the presence of increased dental wear as assessed by the modified O’Brien erosion index (15), which confirms the seriousness of the consequences of the most common oral habit in patients with RS, i.e., bruxism.

In conclusion, the present study of a large population of patients with RS indicates that such individuals have very characteristic and early oral habits consisting particularly of diurnal bruxism but also stereotyped tongue movements and oral breathing. The oral manifestations are also characteristic (drooling, high-arched palate and anterior open bite), with a lesser prevalence of caries than in the non-mentally disabled population, and greater periodontal problems. Our efforts therefore should focus on the diagnosis and early correction of the parafunctional habits, promoting restorative treatment, and providing instructions on correct oral hygiene.
